# Effect of Prophylactic Intrathecal Pethidine on the Incidence of Shivering on Mothers Undergoing Cesarean Section Under Spinal Anesthesia: A Randomized Controlled Trial

**DOI:** 10.3389/fmed.2022.887724

**Published:** 2022-07-28

**Authors:** Timsel Girma, Wagaye Alemu, Sofia Assen

**Affiliations:** ^1^Department of Anesthesiology, Dilla University, Dilla, Ethiopia; ^2^School of Public Health, Dilla University, Dilla, Ethiopia

**Keywords:** intrathecal, pethidine, shivering, pruritus, postoperative nausea, vomiting

## Abstract

**Background:**

Shivering is the most common and unpleasant complication of anesthesia with an incidence of 70.7% in cesarean section done under spinal anesthesia which is associated with cardiovascular and respiratory complications. Even though it causes such devastating complications; the prevention of shivering is not well investigated. This study aimed to assess the effect of intrathecal pethidine on the incidence and severity of shivering in patients undergoing cesarean section under Spinal anesthesia.

**Materials and Methods:**

After obtaining ethical clearance double-blinded single centered a randomized controlled trial was conducted in a total of 86 pregnant mothers who were randomly allocated into two groups by computer-generated random number. Approximately 1 ml of 10 mg preservative-free pethidine was added to 12.5 mg of 0.5% bupivacaine for spinal anesthesia in the treatment group and 12.5 mg of 0.5% bupivacaine alone was given in the control group. Incidence and severity of shivering, as well as adverse effect was recorded intraoperatively, in post-anesthesia care unit (PACU) and ward. Independent sample *t*-test, Mann–Whitney *U* test and chi-square were used for analysis. A *p*-value less than 0.05 was considered statistically significant.

**Results:**

Shivering was observed in 53.5 and 20.9% in the control and treatment groups, respectively, which was statistically significant with *p* = 0.002. The risk of developing shivering was reduced by 61% in the treatment group with (*RR* = 0.39 and *CI* of 0.205–0.745); the intensity of shivering was also higher in the control group than in the treatment group with *p* = 0.004. Considering an adverse effect, the incidence of PONV was not significantly different between with *p* > 0.05 while the incidence of pruritus was higher in the treatment group than the control group with *p* = 0.003.

**Conclusion:**

Adding 10 mg of preservative free pethidine intrathecally during spinal anesthesia is effective in reducing incidence and severity of shivering, without causing significant adverse effects on mother.

## Background

In the past few decades, there has been increasing in the rate of cesarean section (C/S) in both developed and developing countries due to maternal, fetal, medico-legal, and social factor. Because of the rising in the rate of C/S more attention should be paid for its outcome due to the high rate of mortality and morbidity. This complication is not occur due to surgical procedure only it can be attributed to preferred anesthetic technique as well as physiological changes which occur during pregnancy and medical condition of the mother (1–7).

The choice of anesthetic technique depends on obstetrics indication, experience of anesthetist, and choice of the mother. The spinal anesthesia (SA) become popular due to its simplicity, fast onset, good sensory & motor block with avoidance of airway obstruction, and risk of aspiration as well as reduced occurrence of deep vein thrombosis (1, 8–10).

Even if SA is considered to be safe it is not complication-free. Shivering is the most common and unpleasant complication of anesthesia which reaches incidence of 40–70% after SA which is associated with deleterious sequela (11).

Shivering is defined as a spontaneous and involuntary rhythmic oscillatory contraction of muscles with a frequency of 4–8 hertz (12, 13). It will occur as a physiologic response to hypothermia and muscular hyperactivity which increases basal metabolic heat production up to 600%; even though muscular activity may be increased in normothermic patients during the perioperative period. This suggests that shivering may be caused by other mechanism rather than heat loss such as pain, uninhibited spinal reflex, reduction in sympathetic activity, adrenal suppression, discharge of fever-causing agents, and respiratory alkalosis (12–19).

Even if the exact cause of SA-induced shivering is unknown; its vasodilatation effect, increased cutaneous heat loss, decreases the threshold of shivering and redistributes the heat from the core to periphery will predispose the patient to shivering (20).

Shivering will be prevented or managed with non-pharmacological and pharmacological treatments. The non-pharmacological method includes forced-air warmers, active & passive cutaneous counter warming, blankets, body core warming, electro-acupuncture, fluid warming, and increasing operating room temperature prevents shivering (21, 22).

From pharmacological agent, Pethidine is gold standard drug that has been used for the long period of time for the prevention and treatment of shivering during surgery and emergence (23).

Pethidine is a synthetic phenylpiperidine derivative opioid that acts on μ and *k* opioid receptors. It has several structural features which mimic local anesthetics including tertiary amine, an ester group, and lipophilic phenyl group. The administration of pethidine intrathecally blocks sodium channel as blocked by lidocaine (24).

Antishivering effect of meperidine is mediated by its effect on *k* opioid receptors (15, 25).

The study aimed to assess the effect of Intrathecal Pethidine on the Incidence and Severity of Shivering during Cesarean section under Spinal anesthesia.

## Materials and Methods

An institutional-based randomized controlled trial (RCT) was employed in 86 pregnant mothers who undergo C/S under SA from September 2021 to January 2022 in Dilla University Referral Hospital.

The ethical clearance was obtained from the institutional review board (IRB) of Dilla University before the beginning of the study. The written informed consent was obtained from each study participant. This study was prospectively registered by October 2021 on pan African clinical trial registry^1^ with the Trial number of **PACTR202110617970132.** The study was reported in line with the 2010 CONSORT guideline for clinical trial http://www.consort-statement.org. In addition, we registered our study on research registry with unique identification number of research registry 7357 https://www.researchregistry.com/browse-the-registry#home

All elective ASA II pregnant mothers scheduled for C/S were included in this study. Patients whose initial axillary temperature < 36 and > 38°C, the one with allergy history to pethidine & LA agent, Patient who received thermoregulatory medication or antipsychotic medication, a patient who has a history of alcohol or substance abuse, cardiovascular disease, preeclampsia, and patient with antepartum hemorrhage were excluded from the study.

As the pregnant mother arrived at to operating room standard monitoring was applied; axillary temperature and vital signs were recorded. The parturient was resuscitated with 20 ml/kg crystalloid which was warmed to 37°. After preparing equipment for SA, the anesthetist opens the envelope and SA was performed with 12.5 mg of 0.5% bupivacaine which was standard approach of the hospital for group C while for group T SA was performed by adding 1 ml of 10 mg preservative-free pethidine to 12.5% mg of 0.5% bupivacaine. The medication was prepared by investigators, coded and the responsible anesthetist gives SA based on the code he/she get inside envelope. In all parturient, SA was performed in sitting position between L3/L4 and L4/L5 through midline approach. After a performance of SA parturient was positioned, the exposed body area was covered with non-warmed drapes & put on nasal oxygen with a flow of 4l/min. A block was assessed by pinprick for sensory & Bromage scale for a motor block. The procedure was started with a sensory level of T6 & Bromage scale > 3 blocks. Axillary temperature and vital signs were monitored every 10 min to the end of surgery. The patient was maintained by warmed fluid throughout the procedure to post anesthesia care unit (PACU). If the systolic BP is reduced by more than 20% & HR drops to less than 50 beats per min IV adrenaline 1 μg/kg and atropine 0.02 mg/kg was given, respectively. Incidence of shivering was recorded intraoperatively & in PACU continuously. Its intensity was determined by using a validated 5 scale of Crossly and Mahajan which defined as **0** no shivering, **I** piloerection or peripheral vasoconstriction but no visible shivering, **II** muscular activity observed only in one muscle group, **III** muscular activity observed in more than one muscle group without generalized shivering, and **IV** all body shivering (26) and followed for 1 h in PACU. In PACU, all patients were covered with warmed cloth up to they were discharged. The patient who develops shivering grade ≥ 3 was given 25 mg of IV pethidine. As secondary outcome, incidence of postoperative nausea & vomiting (PONV) as well as pruritus were recorded & followed intraoperatively & for the first 6 h every 2 h in PACU & ward. Those complications were scored by validated 11 scales of numeric rating scale (NRS). If nausea & vomiting score is ≥ 4, 10 mg metoclopramide was given and if pruritus NRS score is ≥ 4, 25 mg of IV Diphenhydramine was given. Data were collected using pretested and structured checklist by two trained BSc anesthetists who had no any role in the patient management.

For this study, the following descriptions were used:

Incidence of shivering occurrence of spontaneous & involuntary rhythmic oscillatory contraction of muscles during intraoperative time and in PACU and full-fill Crossly and Mahajan scale.

The postoperative nausea and vomiting sensation of urgent to vomit and actual oral passage of GI content once or more than one times within 6 h of postoperative time.

The postoperative pruritus-unpleasant sensation of skin which lead to the desire to scratch within 6 h postoperatively.

### Sample Size and Sampling Technique

The sample size is calculated based on a previous study done in Gondar which showed that the incidence of shivering following C/S under SA was 70.7% (22). We consider the assumption of a 30% reduction in the incidence of shivering in the study groups in comparison to the control group. By using alpha 0.05 & power of 80%, a sample size of 39 in each group is determined.


$$n=\frac{\text{P}1\left(1-\text{p}1\right)+\text{p}2\left(1-\text{p}2\right)*{\left(Z\alpha \backslash 2+Z\beta \right)}^{2}}{{\left(\text{p}1-\text{p}2\right)}^{2}}$$

Where

P_1_ = 0.707

1 - p_1_ = 0.293

P_2_ = 0.407

1 - p_2_ = 0.593

(P_1_ - p_2_) = 0.3


$$\frac{0.707\left(0.293\right)+0.407\left(0.593\right)*{\left(1.96+0.84\right)}^{2}}{{\left(0.3\right)}^{2}}=39$$

With an account of a 10% attrition rate, a total of 86 participants were selected using a systematic random sampling method.

### Blinding and Randomization

Opaque sealed envelope which has a random number generated by computer & code was prepared by the investigator as per the schedule of the day. Before the performing SA, the responsible anesthetist will open the envelope to see random numbers & code found inside. These codes are either labeled as “T” or “C,” where “T” stands for parturient received 12.5 mg of 0.5% bupivacaine & 10 mg preservative-free pethidine prepared in 1 ml by investigator and “C” for the parturient received 12.5 mg of 0.5% bupivacaine alone. The responsible anesthetist had no any role in data collection and interpretation so he was not blind. In this study, the data collector & the patient were blind for what was given.

### Data Analysis and Interpretation

After data collection is completed, data were checked manually for completeness and then coded and entered into the SPSS version 25 computer program for analysis. Normality test was done using Shapiro wilk & histogram as well as homogeneity of variance was done by Levene’s test of equality of variance (age, weight, height, gravidity, parity, preoperative vital sign, and temperature) was normally distributed and an independent sample *t*-test was used for analysis. The severity of shivering, other not normally distributed data was analyzed by the Mann–Whitney *U* test. Incidence of shivering, pruritus, and PONV were analyzed using the chi-square test. The result was presented as mean ± *SD* & median (*IQR*) for continuous variables & number & percent for categorical variables. Risk of developing shivering was assessed by relative risk (RR) with 95% confidence interval (*CI*) where *p*-value of less than 0.05 was considered statistically significant.

## Results

### Demographic and Perioperative Characteristics

**FIGURE 1:**
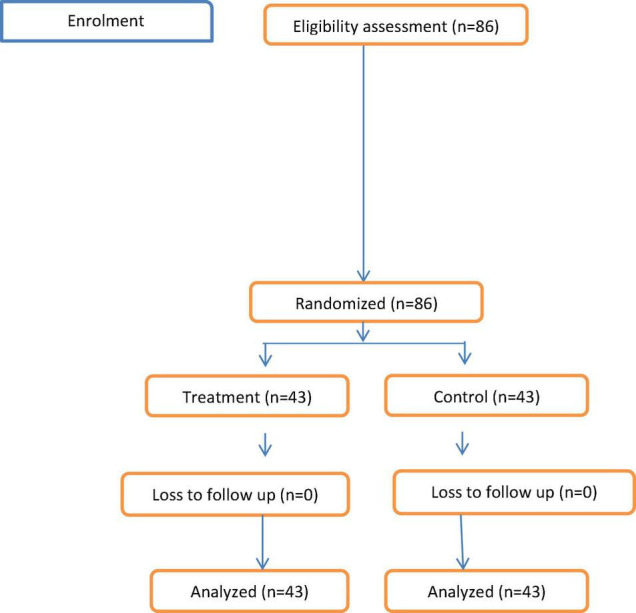
Patient enrollment diagram.

During a study period total of eighty-six, patients were recruited and included for final analysis based on whether they received 10 mg of intrathecal pethidine or not during spinal anesthesia (Figure 1). An independent sample *t*-test was used to compare continuous data of the patients. The result indicated that demographic data were comparable between the two groups with a *p*-value greater than 0.05 (Table 1).

**TABLE 1:** Demographic and perioperative characteristics of pregnant women undergoing C/S under spinal anesthesia (SA) in Dilla University Referral Hospital, 2021/22.

Variables	Treatment	Control	*P*-value
Age	25.9 ± 3.7	27.2 ± 3.5	0.096
Weight	72.5 ± 8.8	70.3 ± 7.9	0.215
Height	164.2 ± 6.3	164.1 ± 5.1	0.956
Gravidity	2.5 ± 1	3 ± 1	0.095
Parity	1.3 ± 1.1	2 ± 1.3	0.095
Baseline MAP	88.6 ± 10.7	87.3 ± 10.1	0.108
Baseline HR	91.3 ± 15.4	86 ± 15	0.536
Baseline temp	36.5 ± 0.21	36.4 ± 0.26	0.244
Baseline Spo2	96.7 ± 1.6	96.8 ± 1.3	0.660
Duration of surgery	44.06 ± 8.46	44.88 ± 7.59	0.640
Total blood loss	545.35 ± 66.23	523.25 ± 66.57	0.127

*Hint all of them were within gestational age of 37 to 42 weeks.*

### Intraoperative Data

**FIGURE 2:**
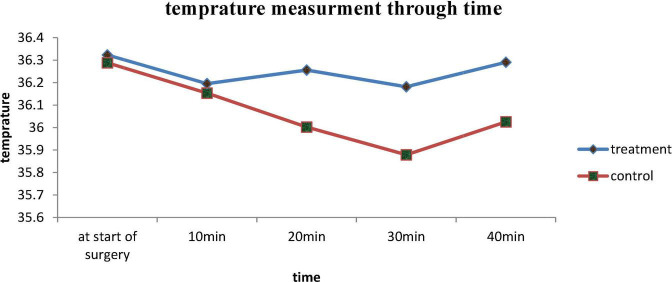
Intraoperative axillary temperature measurement of treatment and control group in Dilla University Referral Hospital, 2021/22.

Intraoperative vital sign was followed from start of surgery to end in every 10 min. In our finding, there was no difference on their vital sign between the groups with *p* > 0.05 (Table 2). In our finding, axillary temperature was reduced through time in the control group while axillary temperature was constant throughout surgery in treatment group which was became statistically significantly different after 20 min with a *p*-value of 0.676, 0.679, 0.013, 0.006, and 0.004 at start of surgery, 10, 20, 30, and 40 min, respectively (Figure 2).

**TABLE 2:** Intraoperative and post anesthesia care unit (PACU) vital sign of pregnant women undergoing C/S under spinal anesthesia (SA) in Dilla University Referral Hospital, 2021/22.

Variable	Treatment	Control	*P*-value
**Intraoperative vital sign**			
MAP at start of surgery	83.9 ± 10.3	84 ± 8.1	0.982
MAP at 10 min	76.8 ± 8.4	75.5 ± 6.7	0.436
MAP at 20 min	74.2 ± 9.9	71.5 ± 6.1	0.138
MAP at 30 min	74 ± 11	74.5 ± 6.9	0.780
MAP at 40 min	77.1 ± 10.6	75 ± 7.4	0.351
HR at start of surgery	87.5 ± 13.1	82.9 ± 13.1	0.113
HR at 10 min	86.3 ± 13.2	83.1 ± 11.9	0.239
HR at 20 min	84.8 ± 11.8	80.2 ± 10.2	0.062
HR at 30 min	84.1 ± 12.9	81.5 ± 10.1	0.315
HR at 40 min	83.5 ± 12.3	82.7 ± 8.3	0.753
**PACU vital sign**			
MAP at arrival of PACU	78.3 ± 9.9	77.4 ± 7.5	0.636
MAP at 30 min	79.7 ± 5.3	81.5 ± 7	0.196
MAP at 60 min	79.5 ± 6.1	81.4 ± 5.7	0.213
HR at arrival of PACU	84 ± 12.3	81 ± 9.6	0.207
HR at 30 min	80.7 ± 10.2	81 ± 9.3	0.904
HR at 60 min	80.7 ± 10	80.4 ± 7.5	0.885

### Incidence of Shivering

**FIGURE 3:**
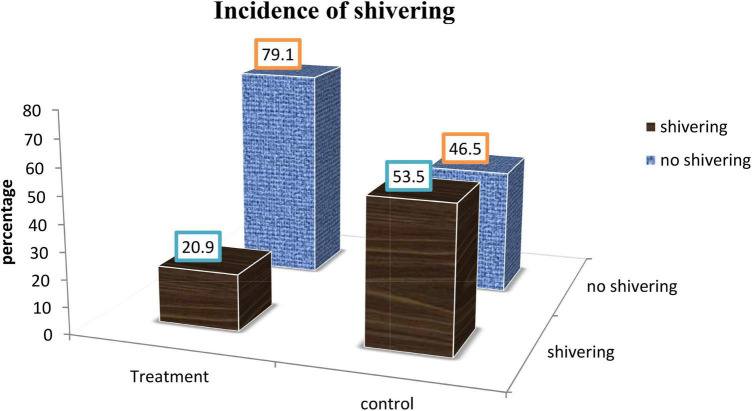
Incidence of shivering between treatment and control group in Dilla University Referral Hospital, 2021/22.

A total of 9 (20.9%) patients from the treatment group and 23 (53.5%) patients from the control group developed shivering which was statistically significant with *p* = 0.002 (Figure 3). The risk of developing shivering was reduced by 61% in treatment group with [*RR* = 0.391, *CI*, (0.205, 0.745)].

### Severity of Shivering

**FIGURE 4:**
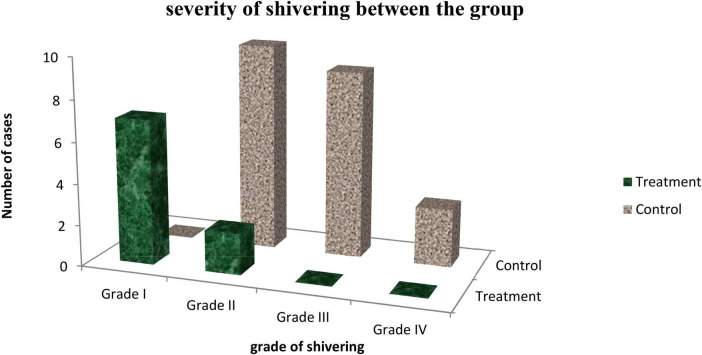
Intensity of shivering between treatment and control group in Dilla University Referral Hospital, 2021/22.

Intensity of shivering was lower in the treatment group than in the control group. Of 23 patients who developed shivering in control group, 4 (9.3%) patients developed grade **IV**, 9 (20.9%) grade **III,** and 10 (23.3%) of the grade **II** none of them develop grade **I** shivering. In the treatment group, 7 (16.3%) of patients developed grade **I** and 2 (4.7%) patients got grade **II** which was statistically significant with *p* = 0.004 (Figure 4).

### Adverse Effect

#### Incidence of Pruritus

As our result demonstrated the incidence of pruritus was 18.6% in the treatment group and 0% in the control group which was significantly different between the group with *p* = 0.003. But none of them needed treatment for pruritus (Table 3).

**TABLE 3:** Incidence of adverse event on pregnant women undergoing C/S under spinal anesthesia (SA) in Dilla University Referral Hospital, 2021/22.

Variable		Treatment	Control	*P*-value
PONV	Yes	11 (25.6%)	13 (30.2%)	0.632
	No	32 (74.4%)	30 (69.8%)	
Pruritus	Yes	8 (18.6%)	0	0.003*
	No	35 (81.4%)	43 (100%)	

#### Postoperative Nausea and Vomiting

The result from the chi-square test detected that incidence of PONV was 25.6% in treatment and 30.2% in the control group, which has no statistical difference with *p* = 0.632, in addition, Mann–Whitney *U* test revealed that the severity of PONV also had no statistically significant difference between the group with *p* = 0.635 (Table 3).

## Discussion

Shivering is troublesome side effect of SA for parturient, anesthetists, and surgeon because of its interference of monitoring and surgery as well as it disturbs patient comfort.

Many classes of substance are responsible to regulation of thermoregulatory center such as biogenic monoamines, cholinomimetics, endogenous peptides, and N methyl D aspartate receptor antagonist. From peptides, antishivering effect of pethidine is mediated by its effect on reducing shivering threshold by two times & its effect on kappa receptors (15, 17, 18).

Our finding demonstrated that the demographic and perioperative characteristic of patients who were enrolled in treatment and control group was comparable with *p* > 0.05. The commonest indication of surgery was previous cesarean section and the mean time for the performance of surgery was 44.1 ± 8.5 min in treatment group and 44.9 ± 7.6 min which was not statistical different with *p* = 0.640. The mean total amount of blood loss in treatment group was 545 and 523 ml in control group with *p* = 0.127 which was comparable. Intraoperative axillary temperature was significantly reduced through time in control group and it became significantly different after 20 min of surgery with *p* < 0.005.

Our study revealed that the administration of 10 mg of pethidine with bupivacaine intrathecally will reduce incidence and intensity of shivering without increasing incidence of postoperative nausea and vomiting. But there was a significant effect on incidence of pruritus.

Our study revealed that the incidence of shivering was 20.9% in treatment group while 53.5% in control group which was statistical significant with *p* = 0.002. The risk of developing shivering was reduced by 61% in treatment group with [*RR* = 0.39 *CI*, (0.205, 0.745)]. Our result is comparable with study done by Shami et al. on 150 parturient had cesarean section. Their finding showed that the incidence of shivering was 50% in control group and 3.6% in pethidine group with *p* < 0.001 (23). In addition, our finding is in line with RCT conducted by J.-Y. Hong and I. H. Lee, Anaraki et al., Abdulzera Sotoodeh Joharmi, and others (25–30).

In our finding, it was clearly seen that the severity of shivering was different between the groups. From 23 patients who developed shivering in the control group 4 (9.3%) patients developed grade IV 9 (20.9%) grade III, and 10 (23.3%) of them grade II while none of them develop grade I shivering. In treatment group 7 (16.3%) of patients developed grade I and 2 (4.7%) patients got grade II which was statistically significant with *p* = 0.004. In agreement to our finding, RCT conducted by savafi et al. showed that the control group develops the higher intensity of shivering than the intrathecal pethidine group with *p* < 0.001 (30). The result of shami et al., Roy et al., J.-Y. Hong, and I. H. Lee, Chun et al., Rastegarian et al., Safavi *et al.*, Nasseri K *et al.*, and Nada and Ezz also comparable to our study and showed that the intensity of shivering was significantly higher in the treatment group (23, 26, 28, 29, 31–34).

In our study incidence of postoperative nausea and vomiting was followed for the 1^st^ 6 h post operatively. The finding showed that the incidence of PONV was not significantly different between treatment and control group *p* = 0.632. In addition, the severity of PONV was also had no statistical significant difference with *p* = 0.635; this finding is in line with result of Rastegarian et al., Nasseri K et al., Shami et al., Roy *et al.*, J.-Y. Hong, and I. H. Lee and others (23, 26, 28, 31, 33).

In contrast to our finding, RCT conducted by Anaraki *et al.* and Khan *et al.* showed that the incidence of PONV was significantly higher in the pethidine group than the control group. The possible explanation for this contrary result is those authors used high dose of pethidine 0.4 mg/kg and 12.5–25 mg, respectively, which is higher than the one we used 10 mg of pethidine (27, 35).

The incidence of pruritus was 18.6% in treatment but none of patients in control group develop pruritus. This finding has statistically significant difference with *p* = 0.003. This finding is in agreement with result of study conducted by Chun et al. which showed the incidence of pruritus was 16% in the pethidine group while it was 0% in the control group (29).

In contrast to our result study done by J.-Y Hong and I. H. Lee indicated that there was no significant difference in the incidence of pruritus between treatment and control group. The possible explanation for this divergence is duration of follow up. We followed the patients for the 1st 6th postoperative hours but they follow them only in intraoperative time (26).

### Strength of the Study

This study was done in resource limited area but as much as possible we tried to maintain randomness of selection regarding time of surgery to avoid time variation between the groups.

### Limitation

One of the limitations of this study is due to financial issues it was not possible to follow the patient for incidence of PONV for more than 6 h. Difficult to control OR and PACU temperature due to there was no temperature adjusting device in our hospital.

We recommend researchers to perform RCT on effect of different dose of pethidine on incidence of pruritus with large sample size.

## Conclusion

Administering 10 mg of pethidine intrathecally during spinal anesthesia is effective in reducing the incidence, and severity of shivering in addition without increasing risk of PONV, while it increases mild incidence of pruritus.

## Data Availability Statement

The original contributions presented in this study are included in the article/supplementary material, further inquiries can be directed to the corresponding author/s.

## Ethics Statement

This study was approved by the Dilla University College of Health Science and Medicine Institutional Review Board with protocol unique number of 005/21-03. The written informed consent for participation in the study was obtained from all patients. All methods were carried out under the Declaration of Helsinki.

## Author Contributions

TG had made substantial contributions to conception, design, analysis, and interpretation of data, acquisition of data, preparing the manuscript of this study, the critical review, and editing of the manuscript drafts for scientific merit and depth. WA and SA made contributions to the interpretation of data, acquisition of data, preparing the manuscript of this study, and the critical review. All authors have read and approved the final version of the manuscript.

## Conflict of Interest

The authors declare that the research was conducted in the absence of any commercial or financial relationships that could be construed as a potential conflict of interest.

## Publisher’s Note

All claims expressed in this article are solely those of the authors and do not necessarily represent those of their affiliated organizations, or those of the publisher, the editors and the reviewers. Any product that may be evaluated in this article, or claim that may be made by its manufacturer, is not guaranteed or endorsed by the publisher.

## Footnotes

1
https://pactr.samrc.ac.za/TrialDisplay.aspx?TrialID=17179

## Abbreviations

C/S: cesarean section
IQR: interquartile range
IRB: institutional review board
MAP: mean arterial pressure
NRS: numeric rating scale
PACU: post anesthesia care unit
PONV: postoperative nausea and vomiting
RCT: randomized controlled trial
SA: spinal anesthesia
SD: standard deviation
